# Clinical Characteristics of Patients With Patent Foramen Ovale and Pyogenic Brain Abscess

**DOI:** 10.1093/ofid/ofag026

**Published:** 2026-01-17

**Authors:** Khalid Abu-Zeinah, Pansachee Damronglerd, Hussam Tabaja, Zachary A Yetmar, Mark J Enzler, Guy S Reeder, Daniel C DeSimone, Larry M Baddour, Omar M Abu Saleh, Cristina Corsini Campioli, Supavit Chesdachai

**Affiliations:** Department of Internal Medicine, Mayo Clinic, Rochester, Minnesota, USA; Division of Public Health, Infectious Diseases, and Occupational Medicine, Department of Medicine, Mayo Clinic, Rochester, Minnesota, USA; Division of Infectious Diseases, Faculty of Medicine, Thammasat University, Pathum Thani, Thailand; Division of Public Health, Infectious Diseases, and Occupational Medicine, Department of Medicine, Mayo Clinic, Rochester, Minnesota, USA; Division of Public Health, Infectious Diseases, and Occupational Medicine, Department of Medicine, Mayo Clinic, Rochester, Minnesota, USA; Department of Infectious Disease, Cleveland Clinic Foundation, Cleveland, Ohio, USA; Division of Public Health, Infectious Diseases, and Occupational Medicine, Department of Medicine, Mayo Clinic, Rochester, Minnesota, USA; Department of Cardiovascular Medicine, Mayo Clinic, Rochester, Minnesota, USA; Division of Public Health, Infectious Diseases, and Occupational Medicine, Department of Medicine, Mayo Clinic, Rochester, Minnesota, USA; Department of Cardiovascular Medicine, Mayo Clinic, Rochester, Minnesota, USA; Division of Public Health, Infectious Diseases, and Occupational Medicine, Department of Medicine, Mayo Clinic, Rochester, Minnesota, USA; Department of Cardiovascular Medicine, Mayo Clinic, Rochester, Minnesota, USA; Division of Public Health, Infectious Diseases, and Occupational Medicine, Department of Medicine, Mayo Clinic, Rochester, Minnesota, USA; Division of Infectious Diseases, Mayo Clinic Health System, Mankato, Minnesota, USA; Division of Public Health, Infectious Diseases, and Occupational Medicine, Department of Medicine, Mayo Clinic, Rochester, Minnesota, USA; Department of Cardiovascular Medicine, Mayo Clinic, Rochester, Minnesota, USA

**Keywords:** bacteremia, brain abscess, echocardiography, patent foramen ovale

## Abstract

Patent foramen ovale (PFO) has a prevalence of approximately 25%. Its association with pyogenic brain abscess (PBA) is unclear. We reviewed adults with PBA and known PFO status from 1 January 2009 through 31 December 2021 at Mayo Clinic. High PFO prevalence (40.5%) was seen. PBA patients with PFO had distinct abscess characteristics but unchanged 1-year, all-cause mortality.

## Graphical Abstract

**Graphical Abstract:**
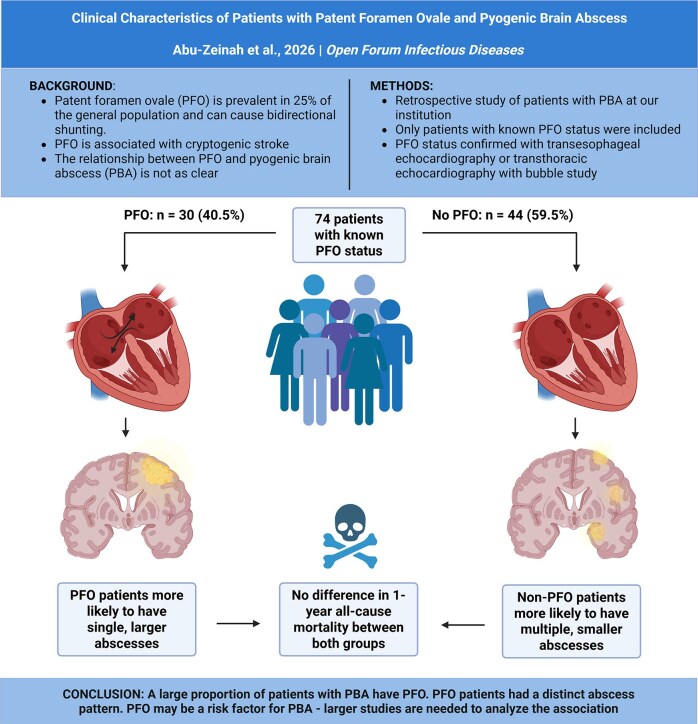
Created in BioRender [1].

Patent foramen ovale (PFO) is the most prevalent congenital cardiac anomaly persisting after birth [2]. Reported prevalence varies by population and diagnostic method, from 14% using transthoracic echocardiography (TTE) with agitated saline (“bubble study”), to 23.7% using transesophageal echocardiography (TEE), and 31.3% with transcranial doppler [3, 4]. Autopsy studies report similar prevalence of 27.3% [5].

PFO is the most common cause of right-to-left shunting in the general population [6], but its clinical relevance is debated. PFO is associated with cryptogenic stroke [7], likely from paradoxical embolization during transient right-to-left shunting. Additionally, limited evidence in the form of case series suggests an association between PFO and pyogenic brain abscesses (PBAs), although this relationship remains poorly understood [8–10]. PBAs are focal, suppurative infections of brain parenchyma arising from contiguous spread or hematogenous dissemination, or classified as cryptogenic when no source is identified [11].

In a similar mechanism to causing cryptogenic stroke, PFO has been hypothesized to increase risk of PBA. Right-left-shunting may allow bacteria and septic emboli from the venous system to bypass the pulmonary circulation [12], which normally traps and clears circulating bacteria via resident phagocytes [13], and reach the brain. Indeed, right-to-left shunting in other conditions, such as pulmonary arteriovenous malformations and cyanotic congenital heart disease (CHD), is a known risk factor for PBA, with 25%–46% of patients with unrepaired cyanotic CHD developing brain abscesses [14–17].

This study aimed to assess the prevalence of PFO in patients with PBA at our institution and to compare clinical characteristics, management, and outcomes of PBA in patients with and without a PFO. Identifying associations between PFO and PBA may improve understanding of PBA risk factors, inform when targeted echocardiographic evaluation in PBA can be considered, and prompt clinicians to consider PBA as a differential diagnosis in patients with PFOs presenting with compatible neurologic symptoms.

## METHODS

We retrospectively reviewed adult patients (≥18 years) diagnosed with PBA between 1 January 2009 and 31 December 2021, at all Mayo Clinic sites (Rochester, Scottsdale, and Jacksonville). Only patients who underwent TEE or TTE with agitated saline (bubble) study were included. TTE without bubble study was excluded due to lower sensitivity for PFO detection [7]. Minnesota residents declining Minnesota research authorization were excluded. Patients were identified using *International Classification of Diseases, Ninth Revision* and *Tenth Revision* codes (324, 098.89, 006.5, G06.0, A54.82, A06.6) for brain abscess. The detailed search procedure and definitions were described in a previous study [18]. *Nocardia* and fungal brain abscesses were excluded, due to distinct epidemiology, host factors, and course compared with typical PBA [11]. Data abstracted from electronic medical records was stored in REDCap [19, 20].

The study was granted exempt status by Mayo Clinic Institutional Review Board (IRB 20-009299) and conducted per Helsinki Declaration guidelines. Informed consent from patients was waived as we used de-identified, retrospective data.

Descriptive statistics were used and reported as median (interquartile range [IQR]) for continuous variables and count (percentage) for categorical variables. The χ^2^ test or Fisher exact test was used for categorical variables and Kruskal–Wallis rank-sum test for continuous variables. One-year survival was assessed using Kaplan–Meier analysis. Analyses were performed using R version 4.2.2 software (R Foundation for Statistical Computing, Vienna, Austria).

## RESULTS

### PFO Proportion

A total of 222 patients developed PBA during the study period. Of these, 74 patients (33.3%) had known PFO status and comprised the study cohort (Supplementary Figure 1). Compared to patients with unknown PFO status, those in the study cohort more frequently had bacteremia and cryptogenic or odontogenic sources of infection (Supplementary Table 1); however, there was no difference in 1-year all-cause mortality between the groups (Supplementary Figure 2).

PFO was detected in 30 of the 74 patients (40.5%). Among those with PFO, intracardiac shunting was demonstrated in 22 patients (73.3%): 12 (54.5%) with right-to-left, 6 (27.3%) with bidirectional, and 4 (18.2%) with left-to-right shunting.

Most patients (n = 52 [70.3%]) underwent echocardiography within 30 days of PBA diagnosis, prompted by the PBA diagnosis itself, suspected infective endocarditis (IE), or bacteremia. Eight (10.8%) patients underwent echocardiography prior to diagnosis, and 14 (18.9%) >30 days after diagnosis (for other indications like valvular assessment, suspected IE, and cryptogenic stroke workup).

### Baseline and Clinical Characteristics

Table 1 demonstrates demographic and clinical characteristics of the study cohort. The median age was 55.5 years; 25 (33.8%) were female. Baseline demographics were similar between PFO and non-PFO groups.

**Table 1.** Baseline Demographics and Clinical Characteristics of Patients With Pyogenic Brain Abscess With and Without Patent Foramen Ovale

Characteristic	Total (N = 74)	No PFO (n = 44)	PFO (n = 30)	*P* Value
Age, y	55.5 (43.2–66.5)	51.5 (42.5–63.5)	59.0 (46.0–67.8)	.288^a^
Female sex	25 (33.8)	14 (31.8)	11 (36.7)	.665^b^
White race	70 (94.6)	41 (93.2)	29 (96.7)	.515^b^
Comorbidities				
Diabetes mellitus	18 (24.3)	13 (29.5)	5 (16.7)	.205^b^
Chronic kidney disease	13 (17.6)	10 (22.7)	3 (10.0)	.158^b^
Heart failure	19 (25.7)	14 (31.8)	5 (16.7)	.143^b^
Active malignancy	7 (9.5)	3 (6.8)	4 (13.3)	.347^b^
Prior stroke	8 (10.8)	3 (6.8)	5 (16.7)	.180^b^
Immunosuppressive therapy	4 (5.4)	2 (4.5)	2 (6.7)	.692^b^
HSCT	1 (1.4)	1 (2.3)	0 (0.0)	.406^b^
Hypertension	21 (28.4)	12 (27.3)	9 (30.0)	.798^b^
Peripheral vascular diseases	23 (31.1)	15 (34.1)	8 (26.7)	.498^b^
Dementia	3 (4.1)	2 (4.5)	1 (3.3)	.795^b^
COPD	11 (14.9)	6 (13.6)	5 (16.7)	.719^b^
Connective tissue disease	4 (5.4)	1 (2.3)	3 (10.0)	.149^b^
Charlson Comorbidity Index score	4.0 (2.0–6.0)	4.0 (2.0–6.0)	4.0 (2.0–7.8)	.748^a^
Positive blood culture at the time of diagnosis	29 (39.2)	22 (50.0)	7 (23.3)	.**021**^b^
Organisms in blood culture				
*Staphylococcus aureus*	14 (18.9)	11 (25.0)	3 (10.0)	.106^b^
CoNS	1 (1.4)	1 (2.3)	0 (0.0)	.406^b^
Viridans group *Streptococcus* spp	12 (16.2)	8 (18.2)	4 (13.3)	.579^b^
Group C *Streptococcus* spp	1 (1.4)	1 (2.3)	0 (0.0)	.406^b^
Gram-negative bacteria	2 (2.7)	2 (4.5)	0 (0.0)	.236^b^
Multiple abscesses	25 (33.8)	19 (43.2)	6 (20.0)	.**038**^b^
Largest abscess diameter, mm	19.5 (11.2–30.0)	15.5 (10.0–28.0)	25.0 (15.8–37.5)	.**038**^a^
Location of abscess				
Frontal lobe	38 (51.4)	28 (63.6)	10 (33.3)	.**010**^b^
Parietal lobe	24 (32.4)	12 (27.3)	12 (40.0)	.251^b^
Temporal lobe	13 (17.6)	10 (22.7)	3 (10.0)	.158^b^
Occipital lobe	15 (20.3)	11 (25.0)	4 (13.3)	.220^b^
Thalamus	4 (5.4)	1 (2.3)	3 (10.0)	.149^b^
Midbrain	1 (1.4)	0 (0.0)	1 (3.3)	.223^b^
Cerebellum	8 (10.8)	6 (13.6)	2 (6.7)	.343^b^
Brainstem	3 (4.1)	1 (2.3)	2 (6.7)	.347^b^
Potential source of abscess				
Cryptogenic	24 (32.4)	8 (18.2)	16 (53.3)	.**002**^b^
Odontogenic	18 (24.3)	14 (31.8)	4 (13.3)	.069^b^
Infective endocarditis	16 (21.6)	11 (25.0)	5 (16.7)	.393^b^
Seeding from distant sources^c^	6 (8.1)	5 (11.4)	1 (3.3)	.214^b^
Sinusitis	3 (4.1)	3 (6.8)	0 (0.0)	.144^b^
TBI including subdural and epidural spreading	3 (4.1)	3 (6.8)	0 (0.0)	.144^b^
Postcranial surgery	2 (2.7)	0 (0.0)	2 (6.7)	.083^b^
Otitis or mastoiditis	1 (1.4)	0 (0.0)	1 (3.3)	.223^b^
Thrombophlebitis of neck vein	1 (1.4)	0 (0.0)	1 (3.3)	.223^b^
Positive culture from abscess	50 (67.6)	27 (61.4)	23 (76.7)	
Monomicrobial	35 (70.0)	18 (66.7)	17 (73.9)	.167^b^
Polymicrobial^d^	15 (30.0)	9 (33.3)	6 (26.1)	.577^b^
Organisms in abscess culture				
*Staphylococcus aureus*	5 (6.8)	3 (6.8)	2 (6.7)	.980^b^
CoNS	2 (2.7)	1 (2.3)	1 (3.3)	.782^b^
Viridans group *Streptococcus* spp	37 (50.0)	21 (47.7)	16 (53.3)	.636^b^
*Enterococcus* spp	2 (2.7)	1 (2.3)	1 (3.3)	.782^b^
*Actinomyces* spp	4 (5.4)	3 (6.8)	1 (3.3)	.515^b^
Gram-negative bacteria^e^	5 (6.8)	3 (6.8)	2 (6.7)	.980^b^
Anaerobes	15 (20.3)	8 (18.2)	7 (23.3)	.588^b^

Data are presented as median (interquartile range) or count (%).

Bold indicates significant values.

Abbreviations: CoNS, coagulase-negative staphylococci; COPD, chronic obstructive pulmonary disease; HSCT, hematopoietic stem cell transplant; PFO, patent foramen ovale; TBI, traumatic brain injury.

^a^Kruskal–Wallis rank-sum test.

^b^Pearson χ^2^ test.

^c^Presumed hematogenous spread from a noncardiac extracranial infection (eg, pulmonary, intra-abdominal, or skin/soft tissue), in the absence of infective endocarditis.

^d^Polymicrobial abscess was defined as isolation of 2 or more organisms from the abscess culture. Polymicrobial infections most often consisted of *Streptococcus anginosus* with anaerobes (eg, *Cutibacterium/Propionibacterium*, *Prevotella*, *Parviomonas micra*, *Finegoldia*, *Fusobacterium* spp).

^e^Gram-negative organisms identified in abscess cultures included *Pseudomonas aeruginosa*, *Escherichia coli*, *Aggregatibacter* spp, and *Eikenella* spp.

Bacteremia at time of diagnosis was less common in the PFO group compared to the non-PFO group (23.3% vs 50.0%, *P* = .021). The presence of multiple abscesses was less common in the PFO group (20.0% vs 43.2%, *P* = .038). Median abscess diameter was larger in the PFO group (25.0 mm vs 15.5 mm, *P* = .038).

Frontal lobe abscesses were more common in the non-PFO group (63.6% vs 33.3%, *P* = .010). The most common PBA etiology was cryptogenic (n = 24 [32.4%]) followed by odontogenic (n = 18 [24.3%]) and IE (n = 16 [21.6%]). Cryptogenic abscesses were more frequent among PFO patients (53.3% vs 18.2%, *P* = .002).

Diagnostic procedures were performed in 54 (73.0%) patients: 30 (40.5%) underwent stereotactic aspiration and 24 (32.4%) underwent craniotomy with aspiration. Of those 54 patients, 50 (92.6%) had positive abscess cultures, most often monomicrobial. Viridans group streptococci were most commonly implicated (n = 37 [50%]). Among culture-positive cases, pathogen distribution did not differ between groups. Of the 20 patients who did not undergo diagnostic procedures, 19 (95%) had positive blood cultures. Among PFO patients, comparisons by shunt direction revealed no significant differences in most PBA characteristics, but patients with right-to-left shunting tended to have more frontal lobe abscesses (*P* = .007).

### Management and Outcomes

**Figure 1.**
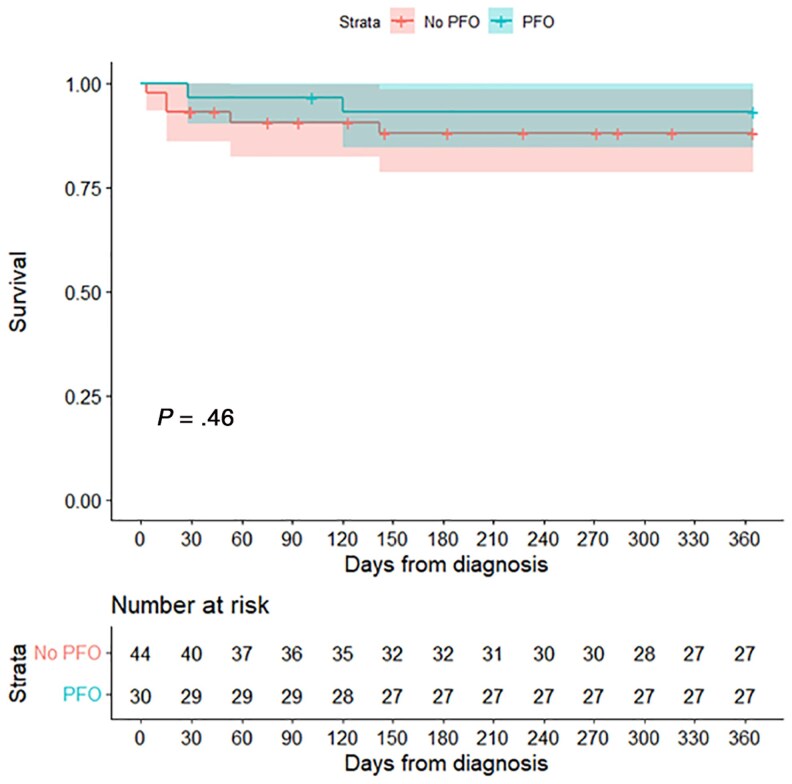
Kaplan–Meier analysis demonstrating no difference in 1-year all-cause mortality between the patent foramen ovale (PFO) and non-PFO groups. Figure generated in R (v4.2.2) [21].

Management strategies were similar between both groups. Thirty-four (45.9%) patients underwent neurosurgical therapy, including aspiration or surgical excision. The remainder were treated with medical therapy. No difference in 1-year all-cause mortality between PFO and non-PFO groups was noted (*P* = .46; Figure 1). Nine patients underwent PFO closure after indentification, typically prompted by a history of stroke or performed concurrently with repair of another structural cardiac abnormality.

## DISCUSSION

In our descriptive study of patients with PBA and known PFO status, 40.5% had PFO. This frequency is higher than prior reports in the general population of 14% using the less sensitive TTE with bubble study [3] and 23.7% using TEE [2, 3]. Although stroke prevalence was similar between the 2 groups, we observed a greater proportion of cryptogenic PBAs in patients with PFO.

PFO patients were more likely to have single, larger abscesses, a pattern consistent with the proposed mechanism of paradoxical embolization, whereby a septic embolus or bacterial inoculum travels through a cerebral arterial territory and leads to solitary abscess. Similarly, PFO-associated strokes have been more frequently described as single cortical infarcts [22]. In contrast, bacteremia, a known risk factor for multiple brain abscesses [23], was more common in the non-PFO group. Given the small absolute number of bacteremic cases and modest absolute differences, these descriptive associations should mostly be considered hypothesis-generating.

Although PFO status did not seem to influence the mortality outcomes or alter management strategies, its importance may instead lie in identifying patients at higher risk of PBA development. Our findings add to the limited literature regarding PBA in patients with PFO. A high frequency of PFO in our study cohort suggests that PFO may be an underrecognized risk factor for the development of PBA. Our study, however, is limited by its retrospective design, descriptive nature, the absence of a matched control group of patients without PBA, and lack of sensitivity analysis. Moreover, not all patients with PBA underwent PFO evaluation with echocardiography. PFO status was therefore not known for most cases, limiting the size of our study cohort, introducing selection bias, and limiting the interpretation of observed differences between the PFO and non-PFO groups. Additionally, our comparison of patients with and without PFO evaluation shows inherent differences between the 2 groups, including in microbiological data. In contrast to our prior institutional cohort in which *Staphylococcus* species predominated [18], *Streptococcus* species were the most frequent abscess isolates in our cohort with PFO evaluation, with minimal contribution from *Staphylococcus* species.

Larger multicenter prospective studies with standardized echocardiographic evaluation of all patients with PBA are needed to determine the true prevalence of PFO in PBA, define the clinical impact of echocardiographic imaging in this scenario, and clarify whether identifying PFO could influence management or outcomes of PBA. At present, no guidelines formally recommend PFO evaluation for all PBA cases. However, similar to the evaluation of cryptogenic stroke, echocardiography can be considered in patients with PBA if no clear source is identified, both to evaluate for PFO and for IE. It is unclear if establishment of an association between PFO and PBA will have definite implications to management. Limited case series have suggested PFO closure for prevention of recurrent brain abscesses [9]. However, current guidelines do not routinely recommend this for PBA, and larger studies are needed to evaluate the utility of PFO closure in prevention of PBA recurrence.

In summary, we observed a higher frequency of PFO in patients with PBA, along with distinct clinical patterns of presentation. Due to the limitations listed, further studies to evaluate the relationship between PFO and PBA are warranted.

## Supplementary Material

ofag026_Supplementary_Data

## Supplementary Data

Supplementary materials are available at *Open Forum Infectious Diseases* online. Consisting of data provided by the authors to benefit the reader, the posted materials are not copyedited and are the sole responsibility of the authors, so questions or comments should be addressed to the corresponding author.
