# Supplementary material for: Clinical Characteristics of Patients With Patent Foramen Ovale and Pyogenic Brain Abscess
Source: Open Forum Infect Dis. 2026 Jan 17;13(2):ofag026. doi: 10.1093/ofid/ofag026 (PMC12862631; doi:10.1093/ofid/ofag026)
Supplement: ofag026_Supplementary_Data [file ofag026_supplementary_data.docx]

**Appendix**

**Supplementary Figure 1 (1)**: Flow diagram of patients with pyogenic brain abscess (PBA) from 2009–2021 and assessment of patent foramen ovale (PFO) status.

**Alt text**: A total of 222 patients with PBA were identified, of whom 134 underwent echocardiographic evaluation. PFO status was determined by transesophageal echocardiography (TEE) or transthoracic echocardiography (TTE) with agitated saline (bubble) study. Patients without echocardiography or without bubble study had unknown PFO status. Bacteremia (yes/no) counts are shown for each subgroup.

**
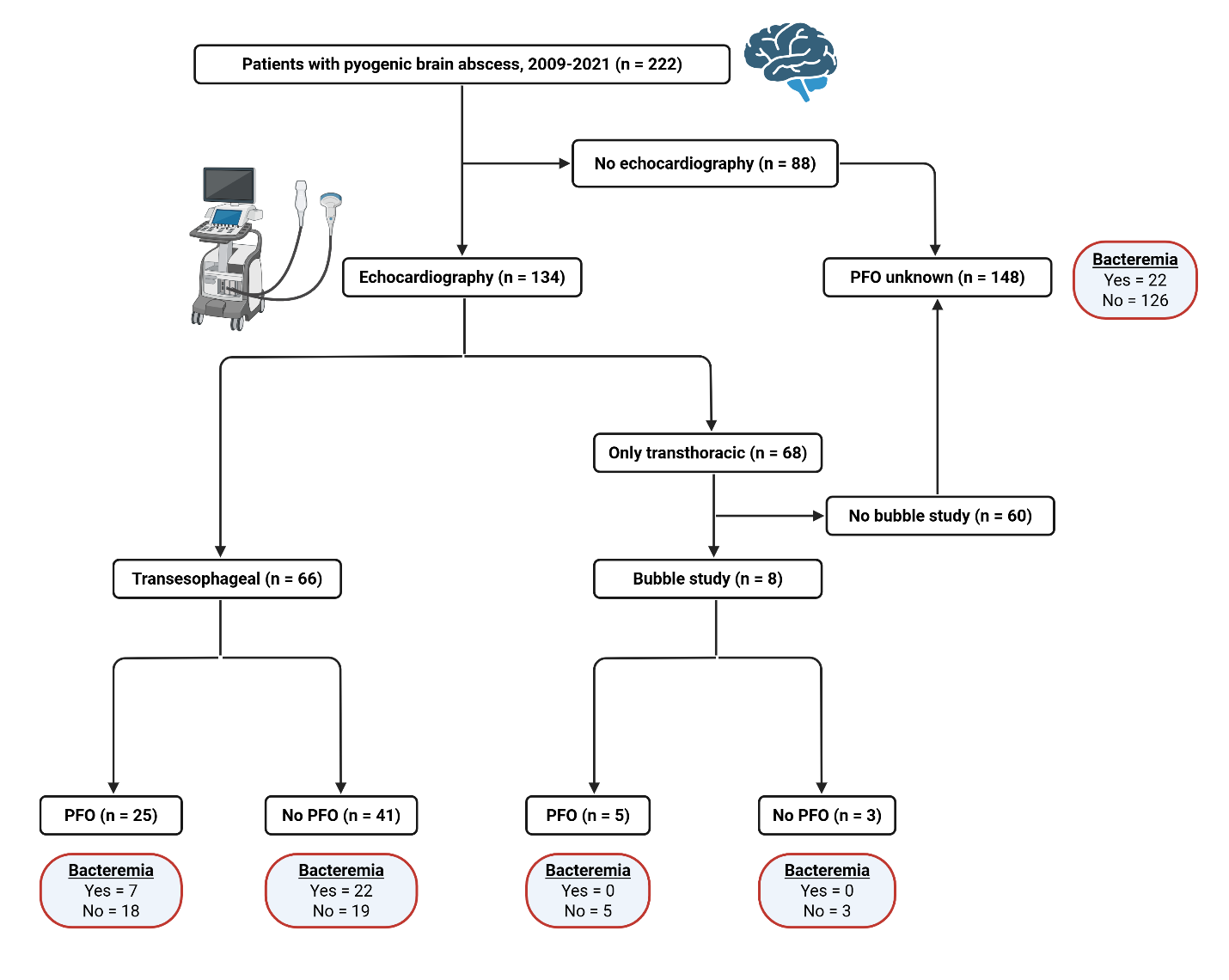
**

**Supplementary Table 1:** Baseline demographics and clinical characteristics of patients with pyogenic brain abscess that underwent PFO evaluation, compared with those that did not.

**Alt Text:**

|  | **Total (N=222)** | **No PFO evaluation (N=148)** | **PFO evaluation (N=74)** | **p-value** |
| --- | --- | --- | --- | --- |
|  | **Median [Interquartile range] or count (%)** | | | |
| **Age, years** | 58.0 (44.0, 67.0) | 59.0 (44.0, 67.0) | 55.2 (43.2, 66.5) | 0.402^1^ |
| **Female** | 80 (36.0%) | 55 (37.2%) | 25 (33.8%) | 0.621^2^ |
| **White** | 194 (87.4%) | 124 (83.8%) | 70 (94.6%) | **0.022^2^** |
| **Comorbidities** |  |  |  |  |
| - **Diabetes mellitus** | 45 (20.3%) | 27 (18.2%) | 18 (24.3%) | 0.288^2^ |
| - **Chronic kidney diseases** | 32 (14.4%) | 19 (12.8%) | 13 (17.6%) | 0.344^2^ |
| - **Heart failure** | 29 (13.1%) | 10 (6.8%) | 19 (25.7%) | **<0.005^2^** |
| - **Active malignancy** | 70 (31.5%) | 63 (42.6%) | 7 (9.5%) | **<0.005^2^** |
| - **Prior stroke** | 15 (6.8%) | 7 (4.7%) | 8 (10.8%) | 0.089^2^ |
| - **Immunosuppressive therapy** | 28 (12.6%) | 24 (16.2%) | 4 (5.4%) | **0.022^2^** |
| - **Hematopoietic stem cell transplantation** | 3 (1.4%) | 2 (1.4%) | 1 (1.4%) | 1.000^2^ |
| - **Hypertension** | 60 (27.0%) | 39 (26.4%) | 21 (28.4%) | 0.749^2^ |
| - **Peripheral vascular diseases** | 44 (19.8%) | 21 (14.2%) | 23 (31.1%) | **<0.005^2^** |
| - **Dementia** | 8 (3.6%) | 5 (3.4%) | 3 (4.1%) | 0.799^2^ |
| - **COPD** | 34 (15.3%) | 23 (15.5%) | 11 (14.9%) | 0.895^2^ |
| **Charlson Comorbidity Index** | 4.0 (2.0, 7.0) | 4.0 (2.0, 7.2) | 4.0 (2.0, 6.0) | 0.363^1^ |
| **Positive blood culture at the time of diagnosis** | 51 (23.0%) | 22 (14.9%) | 29 (39.2%) | **<0.005^2^** |
| **Organisms in blood culture** |  |  |  |  |
| - ***Staphylococcus aureus*** | 22 (9.9%) | 8 (5.4%) | 14 (18.9%) | **<0.005^2^** |
| - **Coagulase-negative *Staphylococcus* species** | 1 (0.5%) | 0 (0%) | 1 (1.4%) | 0.156^2^ |
| - **Viridans group *Streptococcus* species** | 18 (8.1%) | 6 (4.1%) | 12 (16.2%) | **<0.005^2^** |
| - **Group C *Streptococcus* species** | 1 (0.5%) | 0 (0%) | 1 (1.4%) | 0.156^2^ |
| - **Gram-negative bacteria** | 6 (2.7%) | 4 (2.7%) | 2 (2.7%) | 1.000^2^ |
| **Multiple abscesses** | 39 (17.6%) | 14 (9.5%) | 25 (33.8%) | **<0.005^2^** |
| **Largest abscess diameter** | 20.0 (10.8, 30.5) | 20.0 (10.2, 31.5) | 19.5 (11.2, 30.0) | 0.966^1^ |
| **Location of abscess** |  |  |  |  |
| - **Frontal lobe** | 100 (45.0%) | 62 (41.9%) | 38 (51.4%) | 0.182^2^ |
| - **Parietal lobe** | 57 (25.7%) | 33 (22.3%) | 24 (32.4%) | 0.103^2^ |
| - **Temporal lobe** | 57 (25.7%) | 44 (29.7%) | 13 (17.6%) | **0.051^2^** |
| - **Occipital lobe** | 28 (12.6%) | 13 (8.8%) | 15 (20.3%) | **0.0151^2^** |
| - **Thalamus** | 4 (1.8%) | 0 (0.0%) | 4 (5.4%) | **<0.005^2^** |
| - **Midbrain** | 2 (0.9%) | 1 (0.7%) | 1 (1.4%) | 0.615^2^ |
| - **Cerebellum** | 13 (5.9%) | 5 (3.4%) | 8 (10.8%) | **0.026^2^** |
| - **Brainstem** | 6 (2.7%) | 3 (2.0%) | 3 (4.1%) | 0.380^2^ |
| **Potential source of abscess** |  |  |  |  |
| - **Cryptogenic** | 25 (11.3%) | 1 (0.7%) | 24 (32.4%) | **<0.005^2^** |
| - **Odontogenic** | 19 (8.6%) | 1 (0.7%) | 18 (24.3%) | **<0.005^2^** |
| - **Infective endocarditis** | 16 (7.2%) | 0 (0%) | 16 (21.6%) | **<0.005^2^** |
| - **Seeding from distant sources**^3^ | 6 (2.7%) | 0 (0%) | 6 (8.1%) | **<0.005^2^** |
| - **Sinusitis** | 4 (1.8%) | 1 (0.7%) | 3 (4.1%) | 0.074^2^ |
| - **Traumatic brain injury including subdural and epidural spreading** | 3 (1.4%) | 0 (0.0%) | 3 (4.1%) | **0.014^2^** |
| - **Otitis or mastoiditis** | 2 (0.9%) | 1 (0.7%) | 1 (1.4%) | 0.615^2^ |
| - **Thrombophlebitis of neck vein** | 1 (0.5%) | 0 (0.0%) | 1 (1.4%) | 0.156^2^ |
| **Positive culture from abscess** | 178 (80.2%) | 128 (86.5%) | 50 (67.6%) | **<0.005^2^** |
| **Organisms in abscess culture** |  |  |  |  |
| - ***Staphylococcus aureus*** | 40 (18.0%) | 35 (23.6%) | 5 (6.8%) | **0.002^2^** |
| - **Coagulase-negative *Staphylococcus* species** | 2 (0.9%) | 0 (0%) | 2 (2.7%) | **0.045^2^** |
| - **Viridans group *Streptococcus* species** | 64 (28.8%) | 27 (18.2%) | 37 (50.0%) | **<0.005^2^** |
| - ***Enterococcus* species** | 2 (0.9%) | 0 (0%) | 2 (2.7%) | **0.045^2^** |
| - ***Actinomyces* species** | 10 (4.5%) | 6 (4.1%) | 4 (5.4%) | 0.647^2^ |
| - **Gram-negative bacteria** | 24 (10.8%) | 19 (12.8%) | 5 (6.8%) | 0.169^2^ |
| - **Anaerobes** | 45 (20.3%) | 30 (20.3%) | 15 (20.3%) | 1.000^2^ |

1. Kruskal-Wallis rank sum test
2. Pearson’s Chi-squared test
3. Presumed hematogenous spread from a non-cardiac extracranial infection (e.g., pulmonary, intra-abdominal, or skin/soft tissue), in the absence of infective endocarditis.

**Supplementary Figure 2**: Kaplan Meyer analysis comparing one-year all-cause mortality between patients who underwent PFO evaluation (TEE or TTE with bubble study) and those who did not undergo PFO evaluation. P-value = 0.068 by log-rank test.

**Alt text:** Kaplan–Meier survival analysis showing one-year all-cause mortality in patients who underwent PFO evaluation compared to those that didn’t. The survival curves for the group undergoing PFO evaluation (blue) and those without PFO evaluation (red) over the 360-day follow-up period are shown, with overlapping confidence intervals. The number at risk for each group is displayed below the x-axis at regular time intervals. A log-rank test indicates no statistically significant difference in survival between groups (p = 0.068).

**
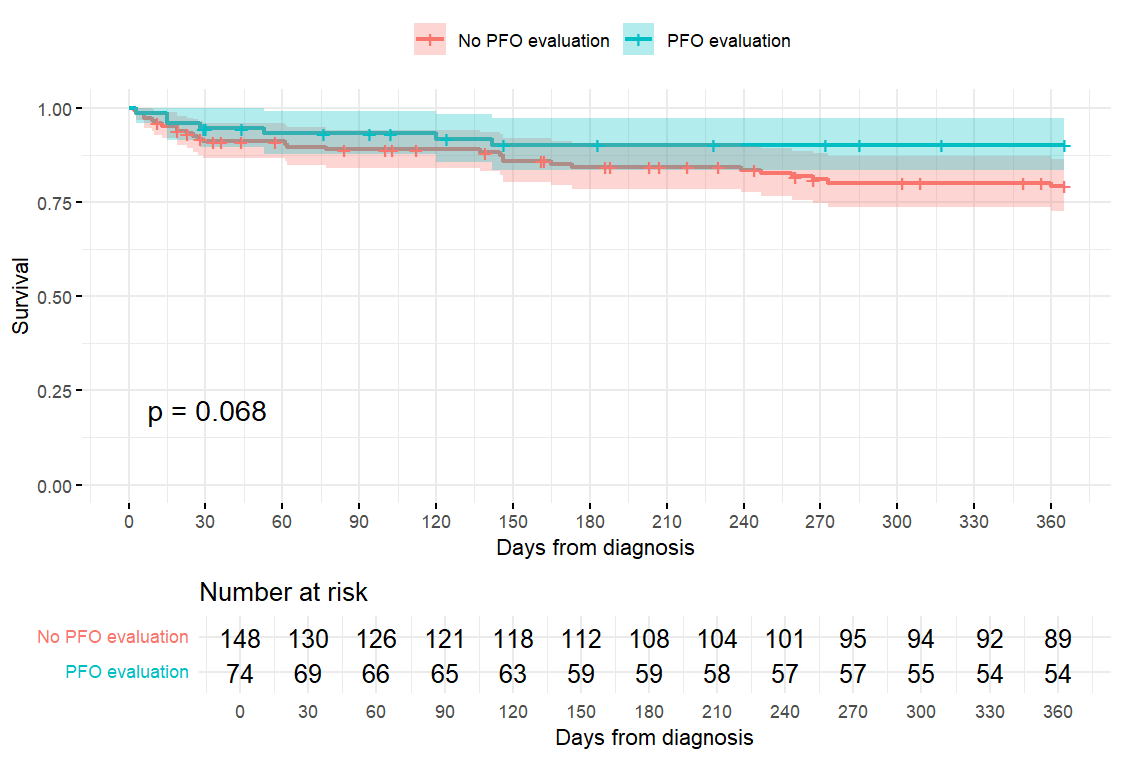
**

**References:**

1. Created in BioRender. Abu-Zeinah, K. (2026) https://BioRender.com/s741j84

2. Posit team (2025). RStudio: Integrated Development Environment for R. Posit Software, PBC, Boston, MA. URL http://www.posit.co/
